# The Dynamic Mechanism on Team Effectiveness in Youth Football: A Chain Mediation

**DOI:** 10.3389/fpsyg.2021.659463

**Published:** 2021-06-28

**Authors:** Juan Li

**Affiliations:** ^1^Shandong First Medical University & Shandong Academy of Medical Sciences, Jinan, China; ^2^School of Economics and Management, Beijing Jiaotong University, Beijing, China

**Keywords:** coach–athlete relationship, team effectiveness, moral leadership, trust, chain mediation, youth athletes

## Abstract

This study aims to deepen our understanding of the relevant research on coach–athlete relationship theory, moral leadership, and team effectiveness theory, and thus explore how to maximize team performance. As such, this study adopts an input-process-output model to explore the effect of coach–athlete relationships on team effectiveness in youth football teams. Participants in this anonymous survey included 312 young athletes, aged 13–19, from professional football schools who filled in questionnaires to provide data on the coach’s moral leadership, team effectiveness, coach–athlete relationships, and trust in the coach. The results indicate that coach–athlete relationships have a significant predictive effect on the moral leadership of coaches, which in turn, has a significant positive correlation with athletes’ trust in coaches; however, coach–athlete relationships have no direct positive correlation with team effectiveness. The coaches’ moral leadership and athletes’ trust in coaches have a chain mediation effect in the impact of coach–athlete relationships on team effectiveness. This study validates the assertion that coach–athlete relationships have a substantial effect on coach leadership. It also refines the coach–athlete relationship theory, provides evidence on the dynamic mechanism in which coach–athlete relationships affect team effectiveness, and enriches team effectiveness theory.

## Introduction

For decades, extensive studies on sports psychology and management have considered coach leadership, coach–athlete relationships, trust, and team effectiveness as key research indicators (Cushion, 2007; Jowett, 2007; Mohammed and Hamilton, 2007; Landy and Conte, 2016). These studies define sports leadership as a complex social process involving a series of interacting elements (Cushion, 2007). Since a social process (Northouse, 2012, p. 3) is a dynamic concept, involving a series of actions and steps (Cambridge Dictionary), scholars posit that sports leadership is also dynamic (Jowett and Arthur, 2019). In recent studies on sports leadership, moral leadership has attracted much attention (Peachey et al., 2015); scholars propose that moral leadership is related to people’s moral cognition and cultural background (Solinger et al., 2020). The moral and cultural background of the Chinese organizational environment has Chinese characteristics; hence, when measuring moral leadership based on the Chinese organizational environment, which is rich in cultural connotations, it is more appropriate to apply localized scales (Sun, 2008).

Based on existing research, one of the indicators of the quality of coaching and sports leadership is the coach–athlete relationship (Jowett and Arthur, 2019). The quality of coach–athlete relationships depends on a dynamic and active process, in which the feelings, thoughts, and behaviors of coaches and athletes are considered. This relationship comprises (1) a sense of intimacy, that is, a positive relationship is established between the coach and the athlete, and the connection is reflected in their mutual trust and respect, emotional care and support, and the love and appreciation in their relationships; (2) commitment refers to the cognitive bond between coaches and athletes, and it is expressed in their willingness to maintain close, long-term connections; and (3) complementarity refers to the behavioral connection between coaches and athletes, and it is manifested in the degree of collaboration and cooperation between leaders and followers (Jowett, 2007).

**Figure 1:**
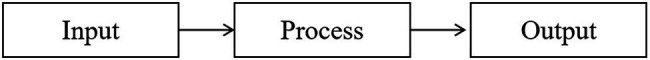
Input-process-output model of team effectiveness.

Chelladurai and Haggery (1991) proposed that team effectiveness affects a sports team’s overall performance and its subsequent behavior. They also proposed that team effectiveness is not easy to conceptualize. Therefore, to understand how to maximize team performance, we adopt the input-process-output model of team effectiveness (Mohammed and Hamilton, 2007; Zhang, 2007; Landy and Conte, 2016; Xu, 2019). In this case, input includes organizational content, team tasks, and team composition (Landy and Conte, 2016; Xu, 2019). The team process includes standardization, communication, collaboration, cohesion, and decision-making (LePine et al., 2008; Xu, 2019). Team output includes productivity, innovation, and team member welfare (Landy and Conte, 2016; Xu, 2019). Moreover, various factors of the team are also dynamic (Toseland et al., 2004). Presently, studies on team-effectiveness also apply this model, or part of it (Carron and Brawley, 2008; Figure 1).

In the Chinese football youth training, most youth football players receive football education and training in boarding professional football schools. These youth football players are the future and hope of the development of Chinese football. This group of individuals has the same characteristics and cultural background of other Chinese organizations. Whether the findings of previous research on coach leadership, coach-athlete relationship, trust, and team effectiveness are consistent with the ones on Chinese football youth training is worthy of further verification. This study explores the dynamic relationship among moral leadership, coach–athlete relationships, trust, and team effectiveness in Chinese youth football teams based on the input-process-output model. Through this study, we aim to deepen our understanding of the relevant research on coach–athlete relationship theory, moral leadership, and team effectiveness theory.

### Research Hypothesis

#### Coach–Athlete Relationship and Moral Leadership

In the literature on coach–athlete relationships and moral leadership, scholars have focused on the influence of leadership on coach–athlete relationships (Schyns and Wolfram, 2008; Hampson and Jowett, 2014), in which the feelings, thoughts, and behaviors of both parties are causally connected (Jowett, 2007). Therefore, coach–athlete relationship can be described further as a two-way process in which coaches and athletes interact with each other (Jowett and Arthur, 2019). Vella et al. (2013, p. 431) proposed that, “Coaching leadership is not a purely behavioral process; it is also an interpersonal influence process, which includes the coach–athlete relationship.” In the field of sports, a few empirical studies combine coach–athlete relationships and coach leadership (Felton and Jowett, 2013; Vella et al., 2013). In the past 30 years, studies have explored the “leader–follower relationship,” and most provide evidence on the influence of a leader’s behavior on the leader–follower relationship (Howell and Hall-Merenda, 1999; Wang et al., 2005). Among them, Hampson and Jowett (2014) verified that a coach’s leadership behavior directly affects the coach–athlete relationship. Therefore, establishing a high-quality coach–athlete relationship is essential for effective and successful coach leadership (Schruijer and Vansina, 2002). To research the potential interaction between the leadership behavior and leadership-subordinate relationship, Graen and Uhl-Bien (1995) and Vella et al. (2010) integrated coach leadership and the coach–athlete relationship. In their study, Jowett and Arthur (2019) proposed that the influence of coach–athlete relationships on coach leadership requires verification. Based on the studies mentioned above, we propose Hypothesis 1.

Hypothesis 1: The coach–athlete relationship positively affects the coach’s moral leadership.

#### Moral Leadership and Team Effectiveness

Leadership is mainly considered as a one-directional process, because the focus, in this context, is more on the coach’s efforts to lead the athlete or team in achieving the predetermined goal (Jowett and Arthur, 2019). When researching team effectiveness, scholars usually consider leadership as an antecedent variable. Among them, Feltz and Chase (1998) pointed out that coach leadership is vital for team effectiveness; Nica (2015) verified that moral leadership has a positive influence on team effectiveness in healthcare organizations; and Ronayne (2004) found a positive relationship between leadership and team effectiveness, which is consistent with the empirical research conclusion by Keshtan et al. (2010).

In contemporary Chinese society, modern (or Western) values and traditional Confucian values coexist, and each set of values has its own moral system (Hwang, 1998; Farh and Cheng, 2000). Similarly, through empirical analysis, the significant correlation between moral leadership and team effectiveness is evident (Cheng et al., 2000, p. 64; Sun, 2008). Based on the above studies, we propose Hypothesis 2.

Hypothesis 2: Moral leadership positively affects team effectiveness.

#### Coach–Athlete Relationship and Team Effectiveness

Jowett stated that coaches and athletes depend on each other, and they cannot achieve nor maintain their best performance individually; therefore, their relationship is the core determinant for an effective and successful coaching (Jowett, 2017), and it creates a social environment in which the interactions between coaches and athletes are either positive or negative. Hence, a good relationship quality between coaches and athletes can maintain the vigor to produce positive effects (Jowett and Arthur, 2019). Empirical evidence indicates that the coach–athlete relationship quality is directly related to the collective effect, although this correlation is extremely limited (Jowett et al., 2012; Hampson and Jowett, 2014). Therefore, the coach–athlete relationship creates a social environment suited for the development of team effectiveness (Jowett, 2008). On this basis, we propose Hypothesis 3.

Hypothesis 3: The coach–athlete relationship positively affects team effectiveness.

#### The Chain Mediation Effect of Moral Leadership and Trust

Hampson and Jowett (2014) indicated that how athletes perceive the coach–athlete relationship determines the collective effectiveness, rather than relying only on coach leadership. Further, Jowett and Chaundy (2004) emphasized that coach leadership and coach–athlete relationship variables should be evaluated simultaneously to predict social cohesion more accurately. In sports teams, both coach leadership and efficient coach–athlete relationships are vital for coaches to build and manage teams with high team effectiveness (Jowett and Arthur, 2019); in this process, both coach leadership and coach–athlete relationship play a synergistic role (Jowett and Arthur, 2019). Concurrently, empirical evidence indicates that the coach–athlete relationship and the coach’s leadership ability are considered simultaneously for better prediction results regarding the sports teams (Jowett and Arthur, 2019). However, existing research on coaching leadership, as a mediating variable between the coach–athlete relationship and team effectiveness, is scarce.

Trust is defined as the belief that the trusted party has relinquished its ability to supervise and control the trusting party, preferring to expose its weaknesses in a risky environment and believing that the other party will not sabotage its own interests (Mayer et al., 1995). In research, trust is usually conceptualized as a key mediation mechanism between leaders and followers (Dirks, 2000), and it has been verified as a mediating variable in the field of sports science. Trust in coach refers to the trust that youth football players have in their coach. Our previous research also verified the mediating role of trust (Li et al., 2021).

Based on the above statement, we propose Hypothesis 4.

Hypothesis 4: Moral leadership and trust have a chain mediation effect between coach–athlete relationships and team effectiveness.

**Figure 2:**
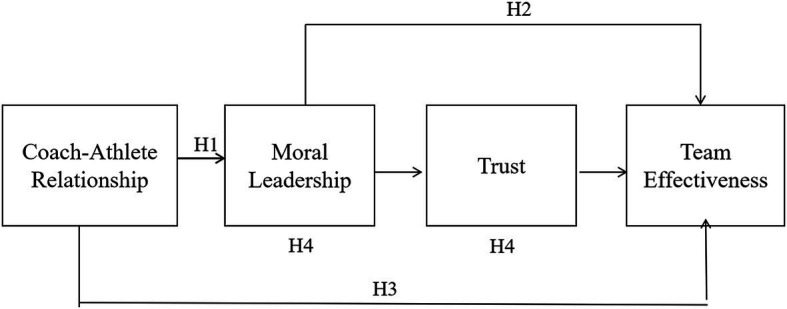
The hypothetical model.

The hypothetical model is shown in Figure 2.

**Figure 3:**
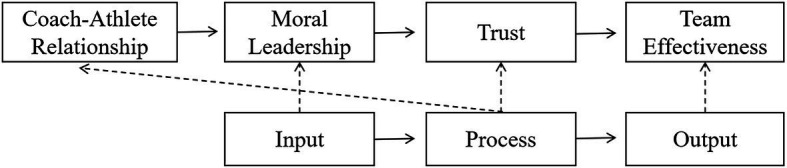
The theoretical framework model of this research.

Next, this study integrates Figures 1, 2 and combines the IPO theory and hypothetical model to obtain the theoretical framework model of this study, as shown in Figure 3. In the theoretical framework of this study, the coach’s moral leadership belongs to the input category, trust belongs to the process category, and the team effectiveness belongs to the output process. In previous studies, the coach-athlete relationship belonged to the process category, but this study will examine the impact of the coach-athlete relationship on moral leadership (input category). This influence is an important verification content of the dynamic mechanism of this study.

## Materials and Methods

### Research Subjects

With the support and authorization of the Ethics Committee of Beijing Jiaotong University (under JG201905017), we conducted a questionnaire-based survey in which youth football players in two professional football schools participated. Except for the two teams that trained and competed in other places, all the youth football players in these two schools participated in the study. The subjects were all male, with an average age of 15.16 years and an age range of 13–19 years. Compared with their peers, the surveyed youth football players are elite athletes. Respondents received professional football training and education in boarding professional football schools. This study issued 312 scales and recovered 296 effective scales (*This study screened the recovered scales and deleted those that did not meet the analysis requirements, such as* scales with one or more unanswered questions, *scales where the same answer was repeatedly selected, etc.*), with an effective rate of 94.87%. Participants were anonymous and were not compensated for the survey; parents/guardians provided the required written informed consent.

### Research Tools

#### Coaching Moral Leadership Scale

This study adopted the Sports Coaching Moral Leadership Scale (Kao, 2000), which includes the following items: *the coach’s knowledge and experience are sufficient to guide me*; *when I make a mistake, the coach corrects me*; *the coach’s life experience guides me*; and nine other items. The results show that the final Cronbach coefficient is 0.83, and the confirmatory factor analysis results are: *χ*^2^/*df* = 3.503, GFI = 0.967, RMSEA = 0.092, RMR = 0.028, CFI = 0.961, NFI = 0.947, and NNFI = 0.936. In other words, the reliability and validity of the scale meet the requirements for further analysis.

#### Coach–Athlete Relationship Scale

This study used the coach–athlete relationship scale by Yang and Jowett (2010). This scale contains four closeness items (including *I like my coach*; *I respect my coach*), with the measured Cronbach’s coefficient as 0.84; three sense of commitment items (including *I feel like being with the coach*, *My sports career is full of hope*), with the measured Cronbach’s coefficient as 0.82; and four complementary items (including *When the coach instructs me, I feel at ease*), with the measured Cronbach’s coefficient as 0.76. The KMO and Bartlett tests were used to verify the validity. The measured KMO value is 0.939, which passed the Bartlett test (*p* = 0.000 < 0.05). The confirmatory factor analysis results are: *χ*^2^/*df* = 3.542, GFI = 0.987, RMSEA = 0.072, RMR = 0.007, CFI = 0.993, NFI = 0.991, and NNFI = 0.987. Therefore, the reliability and validity meet the research requirements (Cameron and Trivedi, 2005).

#### Team Efficiency Scale

This study used the team efficiency table revised by Xu (2019) that contains four self-efficacy items, (including *You are satisfied with your current sports performance*), and the measured Cronbach Coefficient is 0.85; four team performance items (including *The team achieves goals well*; *The team’s training plan is progressing well*), and the measured Cronbach’s coefficient is 0.9; and four team satisfaction items (including *My personal style of the coach* and *The leader is satisfied*), and the measured Cronbach coefficient is 0.81. The KMO and Bartlett test were used to verify the validity. The KMO value is 0.871, and it passed the Bartlett test (*p* = 0.000 < 0.05). The results of the confirmatory factor analysis are: *χ*^2^/*df* = 2.193, GFI = 0.956, RMSEA = 0.063, RMR = 0.051, CFI = 0.98, NFI = 0.964, and NNFI = 0.97. Similarly, the scale has good validity and reliability and can be further analyzed.

#### Trust in Coach Scale

This study uses the revised Scale for Trust in Leader of Dirks (2000), published by Chen and Kao (2006) in the Physical Education Journal as a measurement tool. The original scale is a tool used to measure the trust of the American NCAA basketball team in coaches. It has only one dimension and nine items (including *I believe that the coach can make my performance better and better*), which is suitable for sports teams. The results of the Chinese revision show that it has good validity and reliability and is widely used in the field of sports. The Cronbach’s coefficient measured in this study is 0.934. The KMO value is 0.938. The results of the confirmatory factor analysis are *χ*^2^/*df* = 1.682, GFI = 0.959, RMSEA = 0.059, RMR = 0.023, CF I = 0.989, NFI = 0.972, and NNFI = 0.981. Reliability and validity analysis results show that this quantity is suitable for further analysis.

### Test Process

The researchers distributed and subsequently collected the questionnaires in the school’s classrooms, which they considered as a unit. Prior to distributing the questionnaire to the subjects, they explained the research purpose, that the survey was anonymous and unpaid, and that the questionnaires were to be filled independently. Thereafter, the participants filled in the questionnaires; the entire process took place before class in the afternoon and after dinner and lasted approximately 15 min. We randomly chose the place and time, according to the team’s training time, and excluded the coaches and sports teachers. After data collection, we collated the paper questionnaire data, crosschecked, and archived the paper questionnaires.

## Results and Analysis

### Descriptive Statistics

Correlation analysis was used to research the correlation between age, moral leadership, coach–athlete relationship, trust, and team effectiveness (see Table 1), and the Pearson’s correlation coefficient was used to indicate the strength of the correlation. Specific analysis shows that there is a significant relationship between age and team effectiveness, and the correlation coefficient value is −0.339, which is less than 0. This means that there is a negative correlation between age and team effectiveness. At the same time, the correlation coefficient value is close to 0, indicating that there is no correlation between age and coach–athlete relationship, moral leadership, and trust. Similarly, the coach–athlete relationship is related to trust and moral leadership, and moral leadership is related to trust and team effectiveness. Trust is not related to team effectiveness.

**Table 1:** Variable descriptive statistics.

	M ± SD	Age	CAR	MPL	Trust	TE
Age	15.162 ± 1.915	1				
CAR	4.006 ± 0.651	−0.082	1			
MPL	4.100 ± 0.620	−0.142	0.815^***^	1		
Trust	4.115 ± 0.803	−0.114	0.832^***^	0.816^***^	1	
TE	3.882 ± 0.701	−0.339^***^	0.078	0.121^*^	0.072	1

M, mean; SD, standard deviation; CAR, coach–athlete relationship; MPL, moral leadership; and TE, team effectiveness.

* *p* < 0.05;

*** *p* < 0.001.

### Hypothesis Verification

#### Main Effect

Taking age and coach–athlete relationship as independent variables, and moral leadership as the dependent variable for linear regression analysis, it can be seen from Table 2 that the model formula is MPL = 1.288–0.023*Age + 0.786*CAR, and the model *R* square value is 0.610, which means that alongside age, coach–athlete relationship can explain 61.0% of the changes in moral leadership. When the *F* test was performed on the model, it was found that the model passed the *F* test (*F* = 146.161, *p* = 0.000 < 0.05), which means that age and at least one other item in the coach–athlete relationship have an impact on moral leadership. In addition, the multicollinearity of the model was tested, and it was found that the VIF values are all less than 5, which means that there is no collinearity problem. The D-W value is near 2, which means that the model does not have autocorrelation, there is no correlation between the sample data, and the model is better. The final analysis shows that the regression coefficient value of age is −0.023 (*t* = −1.734, *p* = 0.085 > 0.05), which means that age does not affect moral leadership. The regression coefficient of coach–athlete relationship is 0.786 (*t* = 16.811, *p* = 0.000 < 0.01), which means that coach–athlete relationship will have a significant positive influence on moral leadership. Therefore, Hypothesis 1 is supported.

**Table 2:** Linear regression analysis results.

	DV:MPL		DV:TE	
Constant	1.288^***^ (4.411)	5.957^***^ (15.344)	5.863^***^ (10.067)	6.690^***^ (11.933)
Age	−0.023 (−1.734)	−0.124^***^ (−4.945)	−0.123^***^ (−4.852)	−0.128^***^ (−5.105)
CAR	0.786^***^ (16.811)			−0.162 (−1.802)
MPL			0.019 (0.218)	
*R*^2^	0.610	0.115	0.115	0.130
Adjustment *R*^2^	0.606	0.110	0.106	0.121
*F*	*F* (2,187) = 146.161, *p* = 0.000	*F* (1,188) = 24.457, *p* = 0.000	*F* (2,187) = 12.190, *p* = 0.000	*F* (2,187) = 13.998, *p* = 0.000
D-W	1.979	1.685	1.683	1.717

DV, dependent variable; CAR, coach–athlete relationship; MPL, moral leadership; TE, team effectiveness; and D-W, Durbin-Watson. Values in brackets are *t*.

*** *p* < 0.001.

Then, taking age and moral leadership as independent variables, and team effectiveness as the dependent variable for linear regression analysis, it can be seen from the above table that the model formula is TE = 5.863–0.123*Age + 0.019*MPL, and the model *R* square value is 0.115, which means that depending on age, moral leadership can explain 11.5% of team effectiveness changes. When performing the *F* test on the model, it is found that the model passes this test (*F* = 12.190, *p* = 0.000 < 0.05), which means that age or moral leadership will have an impact on team effectiveness. In addition, the multicollinearity of the model is tested and found that the VIF values are all less than 5, meaning that there is no collinearity problem; and the D-W value is near 2, which means that the model does not have autocorrelation, there is no correlation between the sample data, and the model is better. The final analysis shows that the regression coefficient of age is −0.123 (*t* = −4.852, *p* = 0.000 < 0.01), meaning that age has a significant negative influence on team effectiveness. The regression coefficient of moral leadership is 0.019 (*t* = 0.218, *p* = 0.828 > 0.05), which means that moral leadership will not affect team effectiveness. Therefore, Hypothesis 2 is not verified.

Lastly, taking age and coach–athlete relationship as independent variables, and team effectiveness as the dependent variable for linear regression analysis, as can be seen from the above table, the model formula is TE = 6.690–0.128*age - 0.162*CAR, and the model *R* square value is 0.130, which means that alongside age, coach–athlete relationship can explain the 13.0% change of team effectiveness. When performing *F* test on the model, it is found that the model passes this test (*F* = 13.998, *p* = 0.000 < 0.05), meaning that age and at least one item in the coach–athlete relationship will have an influence on team effectiveness. In addition, the multicollinearity of the model is tested and found that the VIF values in the model are all less than 5, which means that there is no collinearity problem; and the D-W value is near 2, meaning that the model does not have autocorrelation, there is no correlation between the sample data, and the model is better. The final analysis shows that the regression coefficient value of age is −0.128 (*t* = −5.105, *p* = 0.000 < 0.01), which means that age has a significant negative influence on team effectiveness. The regression coefficient of coach–athlete relationship is −0.162 (*t* = −1.802, *p* = 0.073 > 0.05), meaning that coach–athlete relationship will not affect team effectiveness. Therefore, Hypothesis 3 is not verified.

#### Mediation Effect

Following Wen and Ye (2014), the process test for the mediation effect was conducted, and the test results are shown in Table 3. The Bootstrap sampling method was repeated 5,000 times to test the mediation effect. The result showed that the 95% interval of the “CAR⇒MPL⇒TE” mediation path does not include the number 0 (95% CI: 0.260–0.312), indicating that this mediation path exists. The 95% interval of the “CAR⇒Trust⇒TE” mediation path also does not include the number 0 (95% CI: −0.095 to −0.050), which indicates that this mediation path also exists. Next, the chain mediation path was analyzed. The 95% interval of the “CAR⇒MPL⇒Trust⇒TE” mediation path does not include the number 0 (95% CI: −0.075 to −0.040), which indicates that this mediation path exists. Therefore, Hypothesis 4 was verified.

**Table 3:** Mediation effect test.

	MPL	Trust	TE	TE
Constant	1.288^***^ (4.411)	−0.421 (−1.321)	6.690^***^ (11.933)	6.164^***^ (10.582)
Age	−0.023 (−1.734)	−0.004 (−0.316)	−0.128^***^ (−5.105)	−0.120^***^ (−4.842)
CAR	0.786^***^ (16.811)	0.563^***^ (7.324)	−0.162 (−1.802)	−0.362^*^ (−2.275)
MPL		0.577^***^ (7.594)		0.445^**^ (2.813)
Trust				−0.147 (−1.105)
*R*^2^	0.610	0.731	0.130	0.166
Adjustment *R*^2^	0.606	0.727	0.121	0.148
*F*	*F* (2,187) = 146.161, *p* = 0.000	*F* (3,186) = 168.398, *p* = 0.000	*F* (2,187) = 13.998, *p* = 0.000	*F* (4,185) = 9.225, *p* = 0.000
D-W	1.979	2.085	1.717	1.754

MPL, moral leadership; CAR, coach–athlete relationship; TE, team effectiveness; and D-W, Durbin-Watson. Values in brackets are *t*.

* *p* < 0.05;

** *p* < 0.01;

*** *p* < 0.001.

## Discussion

This study uses coach moral leadership and athletes’ trust in coaches as the mediation variables, team effectiveness as the dependent variable, and the coach–athlete relationship as the independent variable to verify the chain-mediating role of moral leadership and trust. The study results indicate that coach–athlete relationships positively affect moral leadership, moral leadership positively affects team effectiveness, coach–athlete relationships do not directly affect team effectiveness, moral leadership, and trust mediate between coach–athlete relationship and team effectiveness, and the path of influence is CAR⇒MPL⇒Trust⇒TE.

### Interpretation and Implications

First, scholars have proposed that the coach–athlete relationship has a positive predictive effect on moral leadership. Jowett and Arthur (2019) mentioned that the quality of the coach–athlete relationship may be vital in determining the effectiveness of coaching, and that it may be a key factor in coach leadership, which may subsequently result in a successful and satisfied team (Jowett and Arthur, 2019). There is substantial evidence regarding the influence of coach leadership on coach–athlete relationships. Based on our research and inferences by scholars, we draw the following conclusions: coach leadership has a positive influence on coach–athlete relationships, and, simultaneously, a good coach–athlete relationship will promote coach leadership behavior; therefore, the coach–athlete relationship and coach leadership are mutually influential and mutually reinforcing. This aligns with Jowett’s concept that coaches who are satisfied with the quality of the coach–athlete relationship are further motivated to maintain their coaching roles (Jowett, 2008).

**Figure 4:**
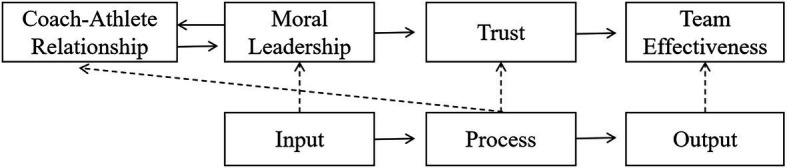
The verified theoretical framework model of this research.

Second, regarding the issue of dynamic influence mechanism, in the integration of the theoretical framework model of this study, it is mentioned that the variable CAR itself belongs to the process category of the IPO model. This study also verified the positive impact of this variable on the moral leadership of coaches. Combining scholars’ research on the impact of coach leadership on CAR and the above conclusions, this study states that, on the one hand, moral leadership will promote the improvement of CAR, and conversely, the improvement of CAR will continue to promote coaches to implement more morals. Leadership behavior goes a step further. More moral leadership behaviors will promote a higher level of CAR and form a virtuous circle, which forms a dynamic process. Thus, the CAR⇒MPL⇒Trust⇒TE process of this study has dynamic characteristics. Therefore, the verified theoretical framework model of this study can be represented as in Figure 4.

Third, evidence from many studies indicates that moral leadership has a positive predictive effect on team effectiveness (Tang and Song, 2009; Wu and Zhu, 2014), which is consistent with the results of this study without age control. However, when age is controlled, moral leaders no longer predict team effectiveness. This is an interesting result, which shows that research on youth football players will present different conclusions due to different age (Li et al., 2021). Although moral leadership has the above empirical performance, it still plays a mediation effect with trust. The moral leadership demonstrated by coaches will win the trust of athletes. This fully illustrates the importance of moral leadership. In the coaches’ education and leadership of youth football players, there is a proverb that “highly learned is a teacher, and only high morality can be a demonstration.” The coach’s personality, conduct, and demonstration are imperceptibly reflected in the day-to-day training, which is particularly important for the growth of youth football players. To summarize our result regarding the coach–athlete relationship and team effectiveness, the coach–athlete relationship is considered to have no direct predictive effect on team effectiveness; however, it affects team effectiveness through the mediation effect of other variables.

Finally, we discuss the chain mediation effect of moral leadership and trust. The moderation effect of leadership has been studied (Sui et al., 2012); however, in our data analysis, moral leadership cannot be characterized as a moderating variable, because, ideally, a moderating variable is related to independent variables and factors in which none of the variables are correlated (James and Brett, 1984; Baron and Kenny, 1986). In this study, moral leadership strongly correlates with coach–athlete relationships (the correlation coefficient is 0.815). Similarly, it was also found that when moral leadership and trust are used as mediating variables, the mediation effect is significant. Therefore, the importance of moral leadership is not only reflected in the interaction with the coach-athlete relationship but also in the ability to mediate the relationship between the coach–athlete relationship and team effectiveness through the athlete’s trust in the coach. This also explains how, in the research by Hampson and Jowett (2014), the coach–athlete relationship and leadership models change at the individual or team level, and how these models affect collective effectiveness.

### Limitations and Implications for Future Research

Future studies should continuously observe and obtain more complete longitudinal data, and thus prove that the coach–athlete relationship and moral leadership can form a closed-loop dynamic relationship for improvement, on a mutual promotion basis, and further promote the outcome variables (such as satisfaction, sports performance, and team effectiveness). Future studies should also expand the sample size and consider integrating a series of related studies to form a dynamic team effectiveness model that includes variables. Further empirical testing is also required to check whether this dynamic relationship exists in other leadership behaviors such as transformational leadership and service-oriented leadership. This study has taken measures to control the common method deviation by using mixed cross-sectional data and relative panel data, and the data samples meet the minimum analysis requirements. However, the sample size is still limited and does not fully represent the overall situation of a youth athlete in China.

## Conclusion

This study proves that in the context of Chinese organizational culture, the coach–athlete relationship has a positive predictive relationship with moral leadership and also verifies the chain mediation effect of moral leadership and trust between coach–athlete relationship and team effectiveness. The CAR⇒MPL⇒Trust⇒TE chain in the input-process-output model of team effectiveness has been verified. Coach–athlete relationships, moral leadership, and trust are reflected in the team input process, and team effectiveness is reflected in the team output process. At the same time, in the prediction of coach–athlete relationship on team effectiveness, since coach–athlete relationship and moral leadership are mutually influencing and promoting each other, there should be a dynamic influence mechanism of moral leadership. Moral leadership is a powerful force. Coaches with this leadership style not only increase team effectiveness in sports teams but also mediate the impact of coach–athlete relationships on team effectiveness based on their learning and improvement capabilities. Further research on the moral leadership of sports team coaches will more effectively contribute to the science of sports organization.

## Data Availability Statement

The raw data supporting the conclusions of this article will be made available by the authors, without undue reservation.

## Ethics Statement

The studies involving human participants were reviewed and approved by the Beijing Jiaotong University (JG201905017). Written informed consent to participate in this study was provided by the participants’ legal guardian/next of kin.

## Author Contributions

JL independently completed this research and fully responsible for this research.

### Conflict of Interest

The author declares that the research was conducted in the absence of any commercial or financial relationships that could be construed as a potential conflict of interest.
